# Effects of lighted incubation and foraging enrichment during rearing on individual fear behavior, corticosterone, and neuroplasticity in laying hen pullets

**DOI:** 10.1016/j.psj.2024.103665

**Published:** 2024-03-15

**Authors:** Saskia Kliphuis, Maëva W.E. Manet, Vivian C. Goerlich, Rebecca E. Nordquist, Hans Vernooij, Frank A.M. Tuyttens, T. Bas Rodenburg

**Affiliations:** ⁎ Animals in Science and Society, Department of Population Health Sciences, Faculty of Veterinary Medicine, Utrecht University, Utrecht, The Netherlands; † Flanders Research Institute for Agriculture, Fisheries and Food (ILVO), Merelbeke, Belgium; ‡ Department of Veterinary and Biosciences, Faculty of Veterinary Medicine, Ghent University, Merelbeke, Belgium; § Adaptation Physiology Group, Department of Animal Sciences, Wageningen University and Research, Wageningen, The Netherlands

**Keywords:** chickens, prenatal, welfare, larvae, chick

## Abstract

Environmental conditions during incubation and rearing can influence stress responsivity of laying hen pullets throughout their lifespan, and therefore have important implications for their welfare. In this study, a 12:12h green LED light-dark cycle during incubation and larvae provisioning as enrichment during rearing were tested as strategies to optimize early-life conditions and thereby decrease stress responsivity in ISA Brown laying hens. A combination of parameters was measured to indicate neuronal, physiological, and behavioral changes that may affect fear and stress. The proteins calbindin D28k (calbindin1), doublecortin (**DCX**), and neuronal nuclein protein (**NeuN**) were quantified after hatch as a proxy for brain plasticity. Plasma and feather corticosterone levels were measured after hatch and at the end of the rearing phase, and fearfulness was investigated through a series of behavioral tests (i.e., voluntary approach, open field, tonic immobility, and manual restraint tests). No effects of light during incubation were found on calbindin1, DCX, or NeuN. Neither of the treatments affected corticosterone levels in blood plasma and feathers. Light-incubated pullets showed less fearfulness towards humans in the voluntary approach test, but not in the other behavioral tests reported in this study. Larvae provisioning had no effect on behavior. Our study showed minor effects of light during incubation and no effects of enrichment during rearing on stress responsivity of laying hen pullets. The small effects may be explained by the enriched rearing conditions for all birds in this experiment (low stocking density, natural daylight, and 24/7 classical music). Given the promising results of lighted incubation in other studies, which were mostly performed in broiler chickens, and evidence regarding the positive effects of enrichment during rearing, the potential of these strategies to improve laying hen welfare needs to be explored further.

## INTRODUCTION

The environmental conditions during the incubation and rearing of laying hens play a crucial role in the development of stress responsivity and the sensitivity to stimuli in later life (Janczak and Riber, 2015; De Haas et al., 2021). Therefore, optimizing incubation and rearing conditions may contribute to better welfare for laying hens. In commercial hatcheries, eggs are incubated in complete darkness. In contrast, incubation under natural conditions involves periodic exposure to daylight when the mother hen leaves the nest during the last days of incubation in search of food (Archer and Mench, 2014a). Due to the embryo's position in the egg at the end of incubation, the right eye is exposed to light from outside the eggshell more than the left eye (Koshiba et al., 2003). This asymmetrical light exposure plays a major role in modulating the development of lateralization of the avian brain, meaning that brain functions are more specialized in either the right or left hemisphere (Rogers, 2008). Several studies – most of which were performed in broiler chicks – have shown that birds with a stronger lateralized brain are less sensitive to stressors, because they are better able to control the fear response initiated by the right hemisphere (Rogers, 2010; Rogers and Kaplan, 2019). The existing evidence also shows inconsistencies with respect to the direction of effects of lighted incubation on behavior, reporting both a reduction (Archer and Mench, 2014b; Archer, 2017) and an increase in fearfulness (Dimond, 1968). The light color used in these studies seems to play a key role in the direction of these reported effects. The use of green light was demonstrated to decrease feather pecking (**FP**) in a study with laying hens (Özkan et al., 2022) and therefore seems to be a promising choice of light color as intervention to improve the welfare of laying hens.

Besides influencing fear-related behaviors, light exposure during incubation has also been shown to affect physiological characteristics. Lighted incubation was associated with reduced plasma corticosterone (**CORT**) in broiler chicks on embryonic day 19 (Özkan et al., 2012) and after a crating challenge at 3 wk of age (Archer and Mench, 2013). Recently published research by Manet et al. (2023a) showed no effects of green light during incubation on hatching characteristics in brown and white laying hens. This means that no negative effects were found either, which is an important prerequisite for application in practice. To our knowledge, no studies have investigated whether lighted incubation affects brain plasticity. Because chicken hatchlings are precocial, they possess a relatively mature brain that enables them to immediately explore their environment independently (Mezey et al., 2012). Postnatal light exposure is associated with neuroplastic changes in the avian retina (Fosser et al., 2013). Therefore, prenatal exposure to light might increase brain plasticity throughout incubation. An improved plasticity is associated with a better adaptive capacity (Arndt et al., 2022), enabling individuals to cope better with stressors and therefore to be less fearful. All in all, existing behavioral, physiological, and neurological evidence suggests that light during incubation has the potential to reduce stress susceptibility and may improve the adaptative capacity of chicks post hatch, leading to better welfare. Calbindin D28k (**calbindin1**), doublecortin (**DCX**), and neuronal nuclei protein (**NeuN**) are 3 proteins that can be used to assess brain plasticity. Calbindin1 has already been associated with neuronal plasticity in the central nervous system (Roussel et al., 2006). Doublecortin is expressed in migrating neurons and therefore also highlights brain plasticity (Capes-Davis et al., 2005). NeuN, in contrast, is considered a marker of mature cells in most vertebrate species including chicks, and therefore highlights neurons that have stopped migrating (Mullen et al., 1992).

After hatching, the rearing environment continues to play an important role in the development of laying hen behavior (Campbell et al., 2019; De Haas et al., 2021). Previous studies demonstrated that enrichment provided during rearing can stimulate brain development and reduce fearfulness (De Haas et al., 2014; Brantsæter et al., 2016; Campbell et al., 2019). One effective environmental enrichment for laying hen pullets could be the provision of live black soldier fly larvae (**BSFL**). From an economic point of view, providing BSFL as a supplement to the diet can result in lower feed cost, without negatively affecting laying hen performance (Lokaewmanee et al., 2023). Additionally, providing pecking enrichment early in life may prevent the development of FP in adult hens (Tahamtani et al., 2016). Previous studies have demonstrated that enrichment with live BSFL has potential welfare benefits in broiler chickens (Ipema et al., 2020) and in adult laying hens (Star et al., 2020; Tahamtani et al., 2021). However, little is known about the effects of live BSFL provided throughout the rearing phase on the behavior of laying hen pullets.

In summary, optimizing incubation and rearing conditions have the potential to alter the responsivity to stressors and to reduce fearfulness, ultimately improving laying hen welfare. We investigated whether light during incubation and BSFL provisioning during rearing decrease fearfulness and CORT levels in plasma and feathers of laying hen pullets. To examine effects on fear behavior, we conducted an array of fear tests [see Kliphuis et al., (2023) for a complete overview]. That paper reported the effects of these early-life interventions on fearfulness, FP, and foraging behavior, all measured on group level (Kliphuis et al., 2023). In the present paper, we report the results of the tests at the individual level, namely voluntary approach, open field, tonic immobility, and manual restraint tests. To assess alterations in the brain, we quantified a selection of brain proteins (calbindin1, DCX, and NeuN) as a proxy for neuroplasticity, and report a behavioral lateralization test. In addition, corticosterone concentrations were measured in feathers (after hatch and at the end of the rearing phase) and in blood plasma (after hatch and during the manual restraint test). Assessing the effects of interventions through a combination of behavior and (neuro-) physiological measures provides a thorough analysis and recognition for the complexity of laying hen development. We expected that pullets exposed to green light during incubation in combination with BSFL provisioning during rearing would be the least fearful and exhibit a lower CORT response to a stressor and a higher brain plasticity compared to control pullets (dark incubation and no larvae enrichment). Because the pullets that received prenatal light exposure may respond differently to the BSFL treatment than dark-incubated pullets, these treatment interactions were also investigated.

## MATERIAL AND METHODS

### Ethics Approval

This study was approved by the Dutch Central Authority for Scientific Procedures on Animals (**CCD**) under license number AVD1080020198685, and by the Animal Welfare Body Utrecht under work protocol numbers 8685-1-01 and 8685-1-03. The experiment is in accordance with the Dutch legislation and the EU Directive on animal experimentation (2010/63/EU).

### Experimental Design

This study was conducted in 2 batches. A 2 × 2 factorial design was carried out that combined light vs dark incubation and presence vs absence of larvae enrichment during rearing. As a result, the following 4 treatments were defined: Dark, no larvae [**DnL**]; Dark, larvae [**DL**]; Light, no larvae [**LnL**]; Light, larvae [**LL**]. Chicks were randomized and housed in 44 pens (20 pens in batch 1; 24 pens in batch 2) without mixing treatments within pens. Due to a randomization error, treatments were unequally divided over pens in batch 1 (3:7:7:3 DnL:LnL:DL:LL). To compensate for this unequal sample size, 4 extra pens were built to increase statistical power in batch 2 (7:5:5:7). A total of 200 pullets per batch were used; thus, the group size differed between batches (10 in batch 1, 8, or 9 in batch 2). When both batches were combined, the total number of pens per treatment was 10:12:12:10. Approximately equal numbers of chicks from incubators with the same light treatment were housed within a pen to avoid incubator effects.

### Incubation Conditions

A total of 1,100 eggs of the layer hybrid ISA Brown (Hendrix Genetics, obtained from hatchery “Het Anker” in Ochten, the Netherlands) were incubated in 2 batches (500 eggs in batch 1 [January 2020]; 600 eggs in batch 2 [April 2021]) at the CARUS research facility of Wageningen University & Research in Wageningen, the Netherlands. Age of the parent stock was 43 wk in batch 1 and 34 wk in batch 2. Eggs in each batch were randomly assigned to either of 2 conditions: Light or Dark incubation. The Light eggs were exposed to 12 h of darkness and 12 h of green light with green LED-strips (520 nm) radiating 400 lux at egg level every day, whereas the Dark eggs were not exposed to light during incubation. Two incubators per condition were used. For more details on the incubation process, see Kliphuis et al., (2023). After hatching, female chicks were health-checked (protocol described in Heijmans et al., (2022) and given a neck label for individual identification. Male chicks and surplus female chicks were culled using cervical dislocation. Chicks were not beak-trimmed.

### Blood and Feather CORT

Immediately after hatching, blood was collected from 40 individuals (5 per sex per incubation treatment and per batch) to measure plasma CORT. Male chicks were included in this part of the study for ethical as well as scientific reasons: males were culled at hatching, and provided extra study material, and sex differences are interesting from a fundamental point of view. The experimenter collected a day-old chick from its incubator and transported it in a foam box to the adjoining dissection room. Within 3 min after opening the incubator, the experimenter decapitated the chick with sharp scissors and collected blood from its neck in an EDTA-coated tube. The samples were stored on ice for a maximum of 5 h. Once all blood samples were collected, they were centrifuged (∼1,200 g) for 5 min (Beckman Coulter Allegra X-15 R), and the plasma was collected and stored at −20°C until analysis. After blood collection, both wings were collected to assess prenatal exposure to CORT as reflected in the down feathers. The wings were stored at −20°C until analysis. In batch 2, CORT levels were investigated in adult feathers as well. Wing feathers 2 and 8 were plucked from 20 hens (5 per incubation per larvae treatments) after culling at 17 wk of age via cervical dislocation, and stored in a dark and dry environment at room temperature until analysis. Plasma CORT levels were assessed using a commercial ELISA kit (*Labor Diagnostika Nord ELISA kit MS E-5400*) (Van Zeeland et al., 2013), following the manufacturer's protocol. Feather CORT was extracted following the detailed protocols available in the supplementary materials (protocols S1 and S2). Briefly, down feathers of both wings were plucked by hand, while the vanes of the adult feathers were cut from the rachis with scissors. The samples were ground using beads and a TissueLyser (Cat. No. 85300, Qiagen). The resulting powder was then extracted in 80% methanol for down feathers (100% methanol for adult feathers) and incubated overnight on an end-over-end roller. The extracts were dried in a Speed Vac Concentrator (CentriVap Concentrator Labconco) at 42°C for 3 h and stored at 4°C until analysis. Extracts were dissolved in buffer of a commercial ELISA kit (c*orticosterone Cayman ELISA kit #501320*) and analyzed according to the protocol of the manufacturer. Due to an error, the down feathers from batch 2 were not analyzed, leaving the sample size at N = 20 (5 per sex per incubation treatment) from batch 1 only. For blood and feathers, absorbance was measured with a microplate reader (DTX880) and concentrations calculated using Anthos Zenyth Multimode Detectors (v.2.0.0.13) and GraphPad (v.7), respectively. Each sample was analyzed in duplicate. If the coefficient of variation between duplicates exceeded 15%, the sample was excluded from the analysis: one dark-incubated male was excluded for the down feathers and 4 dark-incubated females and one dark-incubated male for the blood. The adult feather data were analyzed as corticosterone concentration per feather length to follow other studies (Bortolotti et al., 2009; Jenni-Eiermann et al., 2015), as well as in corticosterone concentration per feather weight to allow easy comparison with the down feather data. Indeed, measuring down feather length was logistically not feasible and not biologically meaningful. For feather 2, 1 individual (LL treatment) was removed from the dataset as visual examination and a chi-square test for outliers showed it to be an outlier in the concentrations per feather length (χ^2^ = 13.914; *P* < 0.001) and per feather weight (χ^2^ = 13.739; *P* < 0.001).

### Brain Calbindin1, DCX, and NeuN

After the chicks were decapitated for blood collection, brains were dissected. The brains were immediately snap-frozen in liquid nitrogen, kept immersed for 8 min, placed in dry ice for a maximum of 4 h, and finally stored at −80°C until analysis. Calbindin1, DCX, and NeuN brain levels were measured using a Western blot technique. More specifically, the protein/GAPDH ratio was quantified. The left hemisphere was weighed in a 2-mL Eppendorf tube and RIPA buffer + protease and phosphatase inhibitor (PP) and 3 beads (ssbeads 3.2 mm) (11079132ss, BioSpec) were added and ground 8 times for 20 s at 30 Hz with Qiagen Tissuelyser II. A protein measurement was performed and adjusted to a final concentration of 200 µg/30 µl. In all the samples, 10 µg/lane proteins were separated by SDS-PAGE on 8–16% Criterion TGX Gel (#5671105; Biorad), under reducing conditions, followed by transfer onto a nitrocellulose membrane (#162-0115; Biorad). The membrane strips were blocked in TBS-Tween (TBST) with 5% low-fat milk, for 1 h at room temperature. The strips were rinsed once in TBST and incubated overnight with 1st antibody (Calbindin1 1:1,000 (#CB28, Swant), DCX 1:1,500 (#ab18723, Abcam), NeuN 1:1,000 (#MAB377, Merck) in TBST + 5% low-fat milk. Strips were washed 3 times for 5 min in 1 × TBST. Then, strips were incubated for 1 h with the antibodies GARPO (1:10,000) (#31460; Thermo) or GAMPO (1:10,000) (#ab6789; Abcam) depending on the host of the 1st antibody in TBST +5% low-fat milk. After washing 4 × 10 min with TBST and 1 × 10 min with TBS on shaker, reactivity was visualized with Supersignal West Dura Substrate (#34075; Thermo) and measured with the programme ImageLab6.1 at Chemidoc MP (Biorad). GAPDH was performed on the same strip after measuring calbindin1, DCX, or NeuN. A soft stripping of 1 h with TBST + 0.02% NaAz was performed, and the same procedure was repeated from blocking, and GAPDH (1:60:000) (60004_1-1g, proteintech) was used as 1st antibody. The bands were quantified with ImageJ. The Western blots took place in the summer of 2022.

### Rearing Conditions

At 1 d of age, 200 female chicks per batch were transported from Wageningen to Utrecht, where they were housed at the Farm Animal Research Facility of the Faculty of Veterinary Medicine at Utrecht University (Utrecht, the Netherlands) throughout the rearing phase, that is, until 18 wk of age. The rearing pens were 246 × 88 × 241 cm (l × w × h), separated by wire mesh and a 60-cm high wooden barrier to prevent visual contact with adjacent pens. The pen floors were initially covered with peat as litter material to create a larger visual contrast with the larvae. However, the peat was replaced with wood shavings from wk 8 onwards in batch 1 and for all of batch 2, after the occurrence of severe eye infections in 2 chicks (Kliphuis et al., 2023). Each pen contained perches, a water bucket with drinking nipples, and a round feeder. Light was provided via vertical high-frequency dimmable bird lights (GlassLux Standard 1 × 36W; Philips, Eindhoven, the Netherlands) and daylight entered the poultry house through skylights with automated hatches (Boon Agrosystems, Barneveld, the Netherlands). In this way, the hours of daylight could be controlled as needed. The number of light hours was reduced from 23 h on d 1 to 12 h at 5 wk of age. Since the experimental batches were conducted in different seasons (February–June 2020 and May–September 2021), daylight control allowed standardization of the light–dark cycle during rearing. Ceramic heat lamps provided a temperature of 35°C on Day 1 and were adjusted in height above the floor over the following days, lowering the temperature in the home pens. Room temperature was gradually lowered from 25°C on d 1 to 18°C at 5 wk of age. A radio played classical music in the poultry house 24/7 throughout the time the pullets were in experimental facilities, to avoid strong responses to environmental noise and entering humans (Davila et al., 2011). Pullets received vaccinations according to a standard Dutch/Belgian vaccination scheme. This was in accordance with the Belgian legislation for commercial egg production, as the chickens would move to the Flanders Research Institute for Agriculture, Fisheries and Food (**ILVO**) in Melle, Belgium, for follow-up research at 19 wk of age. In the first week of life, chicks received standard rearing feed (Starter 1; De Heus, Ede, the Netherlands). After that, new feed was gradually mixed in (see next paragraph).

### Larvae Enrichment

From d 7 onwards, pullets in half of the pens were provided with live BSFL (batch 1: from Circular Organics [Turnhout, Belgium], batch 2: first from Circular Organics, then from Bestico [Berkel en Rodenrijs, the Netherlands] from 6 wk of age onwards, due to delivery problems at Circular Organics). The amount of larvae provided corresponded to 10% of the daily feed intake, as described in the ISA Brown product guide, and therefore increased with age. Tailor-made meal (Research Diet Services, Wijk bij Duurstede, the Netherlands) gradually replaced the standard feed from Day 7 onwards. This feed included added protein and BSFL oil in the diet of the no-larvae pullets (DnL, LnL), to compensate for any nutritional effects caused by the 10% larvae feeding in the DL and LL groups. The larvae were provided in transparent cylinders (15 × 4 cm) with three 9 mm holes each. This design was based on a previous study on broiler chickens (Ipema et al., 2020). A pilot study performed by the authors in December 2019 confirmed that this design was also suitable for laying hen pullets. Two dispensers per pen with fresh BSFL were provided 6 d per week by caretakers during the daily rounds, from 1 to 19 wk of age. On testing days, larvae tubes were always provided 1 h before testing started.

### Behavioral Tests and Observations

Observers who carried out the behavioral tests were blind to the treatments because the tests were carried out in a different room and the pullets were caught by another person. One exception was the tonic immobility test (**TIT**), in which the observers were blind to the incubation but not the larvae condition. Detailed test protocols are available in a repository (Kliphuis, 2023). In addition to the tests described in this paper, the pullets in this experiment were subjected to an array of tests and observations for other experiments that occurred at pen level (novel object test, human approach test, behavioral recovery after a stressor, feather pecking observations, plumage condition, and foraging behavior, described in Kliphuis et al., 2023). To minimize habituation to humans, physical contact with the pullets between behavioral tests was limited as much as possible.

*Detour Test (N = 198).* To assess the effect of lighted incubation and larvae provisioning on behavioral asymmetry, a detour test (**DT**) was performed at 3 wk of age. Pullets were placed in a 61 × 65 × 61 cm (l × w × h) structure containing a transparent Perspex barrier that separated them from a mirror. During 6 consecutive trials, we recorded whether the pullet passed the barrier on the left or right side to approach the mirror. In addition to the visual stimulus (mirror), sound recordings from the home pen were played behind the mirror to provide an auditory social stimulus. The trial ended after 5 min or when the pullet passed the barrier. The chicks were habituated to the apparatus before testing. A trial was considered successful if the pullet passed the barrier. Pullets that passed the barrier once or not at all were removed from the analysis (included N = 198). The laterality index was calculated with the formula (R-L)/(R+L)*100, in which R and L are the number of successful trials the chicken passed on the right and left hand side, respectively (Vallortigara et al., 1999). LnL and LL pullets were expected to have a higher laterality index than DnL and DL pullets, due to the prenatal asymmetrical brain stimulation.

*Voluntary Approach Test (N = 199).* A voluntary approach test (**VAT**) was performed at 6 wk of age to assess fear of humans. The pullet was placed in the far-left corner of an arena measuring 154 × 164 × 100 cm (l × w × h). After 1 min of habituation, the observer entered the arena and crouched down in the near-right corner. The observer extended their hand, holding corn kernels, at floor level. The latency for the bird to approach and eat the feed was scored, with a maximum of 2 min. Time of day was balanced across treatments, to avoid hunger affecting behavior in the test arena. Due to the COVID-19 pandemic, this test was only performed in batch 2 pullets.

*Open Field Test (N = 199).* An open field test (**OFT**) was performed to assess fearfulness at 10 wk of age. The pullet was placed in the middle of an open field arena in the dark. The arena was the same as that used for the VAT. The floor was divided into 25 grids using tape. Once the light was switched on, the duration of freezing and the latency to vocalize and walk were measured, and the number of vocalizations and lines crossed were counted for 5 min. Due to the COVID-19 pandemic, this test was only performed in batch 2 pullets.

*Tonic Immobility Test (N = 383).* We performed a tonic immobility test (**TIT**) at 11 wk of age to assess fearfulness. We placed each pullet on its back in a cradle (40 × 10 × 25 cm [l × w × h]) with its head suspended to the side and held it down with one hand on the sternum and the other gently pushing the head down for 10 seconds. After release, induction was considered successful if the pullet was still in this position after 10 more s. The number of inductions (maximum 3 attempts) and the latency to rise were recorded. The test ended when the pullet rose after a successful induction or after 5 min. Thirteen pullets were excluded due to disturbances during testing.

*Manual Restraint Test (N = 199).* A manual restraint test (**MRT**) was performed at 15 wk of age to assess the behavioral and physiological responses to an acute stressor. Pullets were restrained on their side for 5 min during which the latency and number of vocalizations, and the latency and number of struggles were measured. A blood sample was taken 15 min after the start of the restraint to assess the peak CORT level. This peak sample was collected from half of the chickens in each pen. From the other half, we also took a baseline sample (as soon as possible after catching the bird) and a recovery sample (30 min after the start of the restraint). Due to the COVID-19 pandemic, this test was only performed in batch 2 pullets. Baseline samples were taken as early as possible after catching (earliest to latest sample: 55 to 223 s). Peak and recovery samples were taken 15 and 30 min after the stressor onset, respectively (exact times were recorded). The samples were processed as described in Manet et al. (2023b), and CORT concentrations were determined with ELISA (501320 Corticosterone ELISA kit; Cayman Chemical, Ann Arbor, MI).

### Statistical Analysis

Statistical analyses were performed in R version 4.1.2. All outcome variables were analyzed at the individual level. Incubator (for hatching data) or pen (for all other data) (hereafter, unit) were included as random factors in each model because pullets in the same unit affect each other and are therefore expected to be more alike than pullets from different units. The adult feathers were the only exception, as their sample size per pen was N = 1.

Linear regressions were used for continuous parameters (all physiology parameters). More specifically, linear mixed-effect models were used for the hatching data, and fitting generalized linear models for the adult feather data. A parametric survival analysis was used for the latency parameters (e.g., the latency to start vocalizing), taking censoring into account in cases when data reached the cut-off point. In all cases, the log-normal distribution was selected from several other parametric distributions (Weibull, exponential, Gaussian, logistic, and loglogistic), based on the Akaike information criterion (**AIC**). A generalized linear mixed model (library lme4) was used for count variables (e.g., number of steps in the open field arena), and a negative binomial distribution was always chosen over a Poisson distribution, after visual inspection of the residuals (DHARMa package), to take overdispersion into account. The number of induction attempts during the TIT was analyzed with Bayesian multinomial logistic regression using Stan.

In all models, incubation condition (i.e., green light vs. darkness), larvae condition (i.e., larvae provisioning vs no larvae), and – if performed in both batches – batch were included in the full model as fixed factors, as was incubation × larvae interaction. For the hatching data, sex (i.e., male vs. female) was also included as fixed factor, and incubator and ELISA plate – when applicable – were included as random effects. A backward model selection procedure was followed, using AIC to assess model fit improvement. Factors sex, incubation, and larvae condition always remained in the model to answer the research question regardless of the significance of the effect. Effect sizes were reported as estimates (est.) in case of continuous parameters, hazard ratios (**HR**) in case of latency data, or rate ratios (RR; ratio of mean number in condition *X*/mean number in reference condition) in case of count data, with 95% confidence intervals.

## RESULTS

### Physiological Measurements

Corticosterone levels in plasma, down, and adult feathers 2 and 8 (Figure S1) were not influenced by incubation and larvae treatments, or their interaction (Table 1). DCX showed 2 bands instead of one in the brains from batch 1, possibly due to deterioration. Therefore, only data from batch 2 were included for this protein in the present paper. Calbindin1, DCX, and NeuN levels in the brain after hatching were not influenced by incubation treatment (Table 1, Figure S2). Batch number did not affect corticosterone levels in plasma or calbindin1 levels, but did affect NeuN levels in brain (est. = −0.52, 95% CI [−0.99, −0.06]; *P* = 0.04). Sex had no effect on the reported parameters.

**Table 1:** Summary of measured parameters in chronological order, for each treatment group and for total sample of laying hen pullets. Descriptive data are given as means ± SEM. Effect sizes are given as estimates (est.) in case of continuous parameters, hazard ratios (HR) in case of latency data, or rate ratios (RR, ratio of mean number in condition *X*/mean number in reference condition) in case of count data, with 95% confidence interval.

	Outcome parameter	DnL	LnL	DL	LL	Grand mean	Incubation (dark = ref.)	Larvae (no.larv = ref)
DF	CORTc (ng/mg feather weight)	2.86 ± 1.90	25.12 ± 16.59	NA	NA	10.14 ± 5.07	15.37 [−3.34, 34.07]	NA
Brain	Calbindin1 (/GAPDH ratio)	1.13 ± 0.15	1.03 ± 0.07	NA	NA	1.11 ± 0.06	−0.11 [−0.35, 0.13]	NA
	DCX (/GAPDH ratio)	1.49 ± 0.67	1.16 ± 0.22	NA	NA	1.18 ± 0.18	−0.24 [−0.94, 0.45]	NA
	NeuN (/GAPDH ratio)	1.72 ± 0.33	1.75 ± 0.35	NA	NA	1.29 ± 0.14	−0.01 [−0.48, 0.46]	NA
DT	Laterality index (-100 to 100)	19.70 ± 13.59	−44.44 ± 11.06	−10.91 ± 12.20	−10.88 ± 12.95	−11.74 ± 6.41	**0.73 [0.52, 1.03]**^1^	0.75 [0.54, 1.06]
VAT	Latency to approach (s)	41.17 ± 6.42	34.79 ± 7.14	35.47 ± 6.91	18.30 ± 4.70	31.95 ± 3.15	0.29 [0.09, 0.99]*	0.47 [0.14, 1.63]
	Latency to peck (s)	71.94 ± 6.71	77.41 ± 7.18	83.46 ± 7.00	68.53 ± 6.78	74.53 ± 3.47	0.58 [0.17, 2.03]	1.15 [0.33, 4.02]
OFT	Latency to vocalize (s)	14.58 ± 5.82	32.04 ± 12.99	7.39 ± 1.39	13.11 ± 2.23	16.36 ± 3.33	1.19 [0.63, 2.28]	0.71 [0.37, 1.37]
	Nr. vocalizations	128.16 ± 8.43	134.68 ± 11.37	127.68 ± 10.95	123.06 ± 7.48	127.93 ± 4.62	0.95 [0.79, 1.13]	1.08 [0.91, 1.29]
	Latency to walk (s)	67.62 ± 9.03	63.85 ± 10.92	58.08 ± 9.78	69.35 ± 9.50	65.41 ± 4.87	0.94 [0.71, 1.23]	0.92 [0.70, 1.21]
	Nr. lines crossed	43.89 ± 3.40	47.03 ± 5.04	54.38 ± 6.62	45.94 ± 3.77	47.29 ± 2.27	1.03 [0.81, 1.30]	1.01 [0.80, 1.28]
TI	Latency to rise (s)	97.01 ± 9.97	88.58 ± 8.56	87.91 ± 8.67	91.43 ± 10.57	90.87 ± 4.66	0.80 [0.51, 1.27]	0.83 [0.52, 1.31]
	Nr. inductions	1.39 ± 0.07	1.41 ± 0.06	1.32 ± 0.06	1.57 ± 0.08	1.42 ± 0.03	1.57 [0.99, 2.54]	1.09 [0.69, 1.69]
MRT	Latency to vocalize (s)	26.42 ± 4.01	41.16 ± 10.19	36.89 ± 10.35	37.35 ± 8.91	34.73 ± 4.12	1.12 [0.79, 1.57]	0.97 [0.69, 1.37]
	Nr. vocalizations	75.96 ± 10.65	73.78 ± 14.45	64.40 ± 11.93	68.42 ± 11.74	70.86 ± 5.99	0.89 [0.70, 1.14]	0.91 [0.71, 1.17]
	Latency to struggle (s)	98.96 ± 12.08	83.81 ± 14.71	67.32 ± 12.21	96.00 ± 12.71	88.14 ± 6.50	**0.66 [0.43, 1.03]**^2^	0.64 [0.46, 0.90]**
	Nr. struggles	4.49 ± 0.90	5.05 ± 0.83	4.47 ± 1.61	3.87 ± 0.62	4.43 ± 0.49	1.07 [0.82, 1.40]	0.90 [0.69, 1.18]
	CORTc baseline (ng/mL plasma)	0.328 ± 0.03	0.321 ± 0.05	0.343 ± 0.03	0.324 ± 0.03	0.329 ± 0.02	NA^3^	NA^3^
	CORTc peak (ng/mL plasma)	0.640 ± 0.03	0.700 ± 0.04	0.735 ± 0.05	0.806 ± 0.05	0.722 ± 0.02	1.03 [0.92, 1.16]	1.12 [1.00, 1.26]^T^
	CORTc recovery (ng/mL plasma)	0.551 ± 0.04	0.496 ± 0.03	0.687 ± 0.16	0.687 ± 0.06	0.610 ± 0.04	NA^c^	NA^c^
AF2	CORTc (ng/mg vane)	9282.01 ± 1178.21	7985.23 ± 1000.44	8618.24 ± 1026.49	8772.51 ± 1245.18	8675.33 ± 967.30	−1751.25 [−5692.05, 2189.56]	21.19 [−3919.62, 3962.00]
	CORTc (pg/cm feather length)	69.82 ± 11.37	68.59 ± 18.86	83.90 ± 18.99	48.48 ± 3.13	68.71 ± 7.61	−17.32 [−47.91, 13.26]	2.01 [−32.59, 28.58]
AF8	CORTc (ng/mg vane)	5072.80 ± 230.11	4755.90 ± 191.36	4917.44 ± 266.24	5001.66 ± 234.27	4936.85 ± 201.00	−349.0 [−1162.75, 464.70]	135.6 [−678.12, 949.32]
	CORTc (pg/cm feather length)	36.89 ± 3.63	36.73 ± 1.20	42.94 ± 4.84	35.25 ± 3.53	37.95 ± 1.77	−3.92 [−10.91, 3.07]	2.29 [−4.70, 9.27]

Abbreviations: AF2, adult feather n°2, AF8, adult feather n°8, CORTc, corticosterone concentration, DCX, Doublecortin, DF, down feathers, DL, dark, larvae, DnL, Dark, no larvae, DT, detour test, LL, light, larvae, LnL, Light, no larvae, MRT, manual restraint test, NA, not applicable because larvae provisioning has not started yet, OFT, open field test, TI, tonic immobility test, VAT, voluntary approach test.

1 Interaction between incubation and larvae = 1.36 [0.97, 1.91].

2 Interaction between incubation and larvae = 2.15 [1.17, 3.98].

3 Because sample number (baseline, peak or recovery) was included as fixed factor in the model, only the overall effect sizes were reported. Significant effects are given in bold, *P*-values = ^T^<0.1, *<0.05, **<0.01.

### Behavioral Tests

*Detour Test****.*** Out of 199 pullets, 198 passed the barrier at least twice and were therefore included. Light-incubated pullets tended to be 1.36 times more left-preferring compared with dark-incubated pullets (95% CI [0.97, 1.91]; *P* = 0.08), but this tendency was only present in the pullets that did not receive larvae (Figure S3).

**Figure 1:**
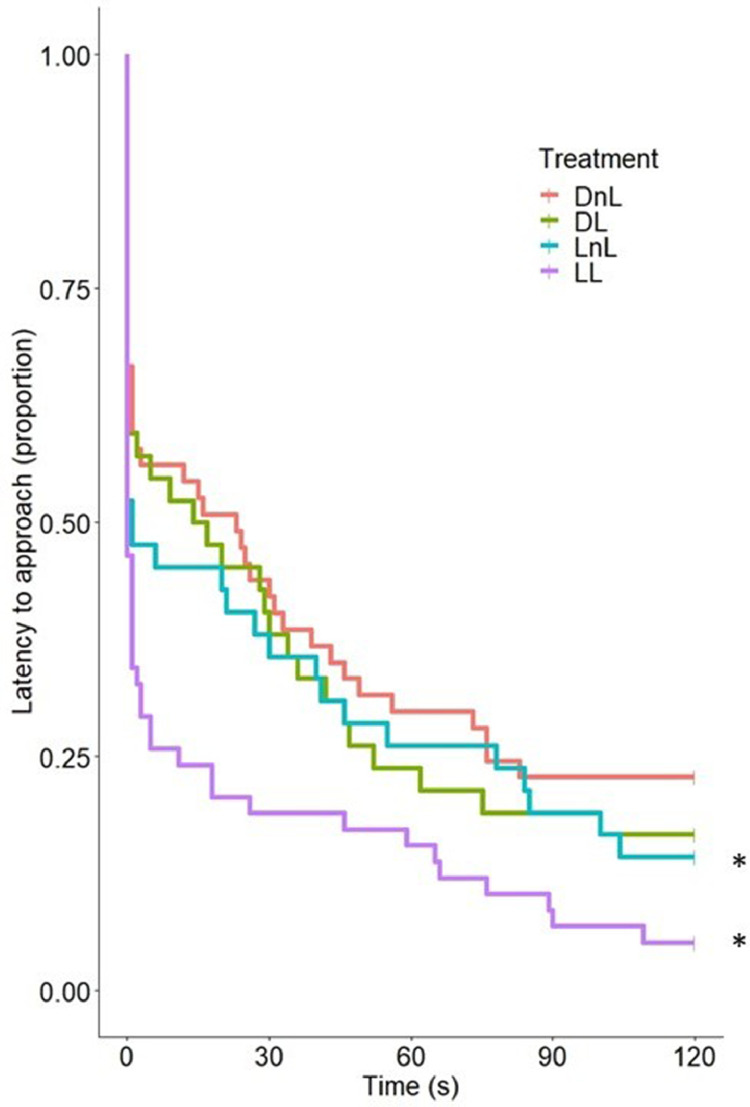
Survival plot of the latency to approach the experimenter in the Voluntary Approach Test (VAT), performed in laying hen pullets at 6 wk of age. Treatment groups: Dark, no larvae (**DnL**); Dark, larvae (**DL**); Light, no larvae (**LnL**); Light, larvae (**LL**). Asterisks indicate treatment groups that differed significantly from the rest.

*Fear Tests (VAT, OFT, TIT, and MRT).* Light-incubated pullets approached 1.29 times faster than dark-incubated pullets in the VAT (95% CI [0.09, 0.99]; *P* = 0.047; Figure 1). Larvae provisioning did not affect the latency to approach. The latency to peck at the bait was not affected by either light incubation or larvae condition (Table 1). Lighted incubation and larvae provisioning did not significantly affect the latency to walk, number of lines crossed, or the latency and number of vocalizations in the OFT (Table 1). Similarly, the treatments did not affect latency to rise in the TIT (Table 1). Treatment did not significantly affect the number of induction attempts, although the pullets from batch 2 needed more attempts to induce TI than the pullets from batch 1 (odds of needing a higher number of inductions in batch 2 = 2.26 (95% CI [1.66, 4.11]). The treatments did not affect the latency or number of vocalizations, or the number of struggles in the MRT. A significant incubation × enrichment interaction was, however, found for the latency to struggle. Pairwise comparisons showed that pullets that received larvae struggled 1.57 times faster than pullets that did not receive larvae (95% CI [1.12, 2.21]; *P* = 0.01), but this effect was only present in dark-incubated pullets (Table 1). Light during incubation did not affect plasma CORT during the MRT (Figure S4). Larvae provisioning tended to increase plasma CORT (est. = 1.12, 95% CI [1.00, 1.26]; *P* = 0.07). There were no significant treatment × sampling time interactions. No correlations were found between behavior during the MRT and plasma CORT (Pearson's coefficient < −0.107; *P* > 0.355).

## DISCUSSION

The present study investigated the effects of a green LED light–dark cycle throughout incubation and BSFL provisioning during rearing on individual stress responsivity and fearfulness in laying hen pullets by measuring indicators in the brain, CORT concentrations, and behavior. Lighted incubation did not affect CORT or brain protein levels determined shortly after hatch. Light-incubated pullets showed a slightly reduced fear of humans in the VAT, but no differences in fearfulness were found in other behavioral tests. Larvae provisioning did not affect any of the reported parameters.

### Corticosterone and Brain Proteins

Our study showed that lighted incubation does not affect plasma or feather CORT shortly after hatching. We expected that opening the incubator to collect the chicks would be stressful and may influence plasma CORT levels. Ericsson et al. (2014) reported that sample collection up to 3 min after a stressor can be considered a reliable baseline, and van der Eijk et al. (2019) showed that CORT peaks at 15 min after a stressor. In our study, the time between the opening of the incubator and blood collection was estimated to be less than 3 min. We are therefore confident that our results are a good estimation of the baseline and peak CORT levels in the chicks. The lack of effect on CORT after hatching might also be explained by Freeman (1982), who found that chicks show a clear but short hyporesponsive period shortly after hatching. A recent study, however, did find changes in day-old chick metabolism due to a combination of lighted incubation and temperature variation (Chelnokova et al., 2023). CORT obtained from feathers can also be a valuable addition to plasma measurements because it provides a retrospective assessment of the avian physiology (Romero and Fairhurst, 2016). CORT in down feathers may reflect the prenatal HPA activity. The blood samples obtained during the MRT indicated no effects of lighted incubation on plasma CORT, which is consistent with our lack of differences found in CORT after hatching. BSFL provisioning tended to increase plasma CORT, which was an unexpected result. One explanation could be that CORT is also associated with higher levels of locomotor activity in birds, and BSFL provisioning may have resulted in more active pullets (Breuner et al., 1998; Breuner, 2000). The absence of significant treatment*sample interactions indicated that lighted incubation and larvae provisioning did not specifically affect baseline, peak, or recovery levels in plasma CORT.

Altogether, no effects of lighted incubation on CORT levels were found in the present study. This contrasts with the results of Özkan et al. (2012), who reported a reduction of CORT 8 h post hatching in broilers after lighted incubation. The difference suggests that broiler physiology may be different from laying hen physiology at an early stage in prenatal development. With regard to protein levels in the brain, light during incubation did not affect calbindin1, DCX, or NeuN levels, indicating that the level of neuroplasticity as measured by these parameters in the chick brains was not affected by the light. We cannot exclude more subtle changes in subtypes of neurons or brain areas that were lost in the whole brain homogenate analyzes we performed, for example the changes in retinal expression of calbindin1 that Fosser et al. (2013) reported.

### Lateralization

In the DT, light-incubated pullets tended to be more left-preferring than dark-incubated pullets. The direction of the tendency was in accordance to our expectations that light-incubated pullets would show a stronger side preference (Rogers, 2008, Rogers, 2010, Manet et al., 2023c). However, this tendency was small and only present in pullets that did not receive larvae. The latter indicates an interaction between both treatments in which the effects of larvae provisioning may have overruled effects of lighted incubation on brain lateralization. Perhaps the benefits of provisioning of a more complex rearing environment (Campbell et al., 2019) overshadow the consequences of prenatal conditions. Mechanisms underlying the effects of lighted incubation and enrichment on brain lateralization, and the apparent interaction between both interventions, need to be explored further.

### Fear Tests

The light-incubated pullets were faster to approach a known human than dark-incubated pullets when tested individually in the VAT. This finding could indicate a decreased fear of humans caused by the light–dark cycle throughout incubation, which is consistent with findings from our recently published study reporting a similar experimental design (Manet et al., 2023b), although the effect of lighted incubation disappeared later in the rearing phase. It is also consistent with a lower stress sensitivity (Archer et al., 2009) and reduced fearfulness (Archer & Mench, 2014b) reported in light-incubated broilers. In contrast, a human approach test performed in the home pen with the same pullets as the current study did not show any treatment effects (Kliphuis et al., 2023). The inconsistency between the pullets’ motivation to approach in both tests may have been caused by the test environment (i.e., in a novel test arena vs the home pen) or by individual vs group-level observations. Another difference between both tests is the offering of a food reward in the individual test, which was not offered in the home-pen approach test. All in all, the reducing effect of lighted incubation on fear of humans seems to be context-dependent. In commercial practice, a reduced fear of humans can improve the chickens’ day-to-day interactions with the farmer. Given that lighted incubation slightly reduced fear of humans in the present study and in that by Manet et al. (2023b), this may be an effective strategy to reduce human-directed fearfulness in commercial practice, albeit only in the first half of the rearing phase.

Treatments did not affect pullet behavior in the OFT, although we did expect light-incubated and larvae-enriched pullets to be more active in the open-field arena, as an indication of reduced fear. Previous studies reported such effects of both lighted incubation and enrichment in similar tests. Broiler chicks incubated with green light vocalized more than chicks incubated with white or red light in a bucket test (Archer, 2017), although this setup mostly indicates motivation for social reinstatement rather than fear of an open arena (Forkman et al., 2007). Environmental enrichment in the form of colored wall drawings and objects was shown to reduce fearfulness in an OFT in individually housed birds (Bryan Jones and Waddington, 1992). However, Tahamtani et al. (2021) found no effects of larvae provisioning on OFT behavior. The OFT is a commonly used test to measure fear, but its outcomes can be difficult to interpret since behavior reflects a complex interplay between fearfulness and motivation for social reinstatement (Forkman et al., 2007). Therefore, a combination of different fear tests might give stronger indications of an effect of treatments on fearfulness. Unfortunately, the results of the present study could not confirm consistent effects across multiple fear tests. One explanation could be the different chick ages at which the tests were performed. Future studies could explore the effect of age on fear response in combination with the treatments reported here.

Contrary to the expectations, lighted incubation and larvae provisioning did not reduce the latency to rise in the TIT. Archer (2017) and Archer and Mench (2014b) found that light-incubated broilers had a shorter latency to rise, indicating reduced fearfulness. The same pattern could not be found in the present study. Moreover, larvae provisioning did not affect fearfulness in this behavioral test.

During the MRT, dark-incubated pullets that received larvae started to struggle faster than dark-incubated pullets that did not receive larvae. Given that the results of the 2 light-incubated treatment groups were in between those of the 2 dark-incubated groups, the interaction is difficult to disentangle. Since this interaction was not found in other parameters of this test, such as latency to vocalize, and it was not present in other behavioral tests in this experiment, there is limited information to further explain this interaction between the incubation and enrichment conditions.

### Summarized Effect of Light During Incubation

The present study showed limited effects of exposure to a green light–dark cycle throughout incubation on behavior, CORT levels, and brain calbindin1, DCX, or NeuN levels in young laying hens. Although the treatment slightly reduced fear of humans, the other behavioral tests performed did not show any significant effects. The finding in this single behavioral test should be put in perspective of all data collected in this experiment. It should also be noted that this lack of difference means that we did not find any negative effects of lighted incubation on behavior. Given the promising results of other studies (Archer, 2017; Archer & Mench, 2014b; Özkan et al., 2022), the incubation conditions should be further optimized to explore the potential to improve laying hen welfare. These and additional behavioral tests were performed in our recently published work studying the effect of green light throughout incubation in 2 different laying hen hybrids, namely ISA Brown and Dekalb White (Manet et al., 2023b). That study, together with the present study, provide a valuable contribution to knowledge on what effects on behavior and physiology could and could not be achieved with light during incubation in laying hen pullets. These studies can be used to fine-tune parameters such as light intensity, duration, and color used throughout incubation in future studies.

### Summarized Effect of Larvae Provisioning During Rearing

This experiment was the first to report the effects of supplying BSFL as enrichment throughout the rearing phase of laying hens. The outcomes showed no significant effects of larvae as enrichment on behavior or CORT levels. Existing literature has been clear about the positive effects of enrichment on reduction of fearfulness (reviewed by Campbell et al., 2019). One possible reason why we did not find such effects could be that perhaps not all pullets were eating the larvae. A recent study reported large individual variation in larvae consumption by laying hens (Tahamtani et al., 2021). This finding indicates that the larvae dispensers in the present study might not be considered enrichment for each individual bird. Although we did not systematically observe interactions with the enrichment, anecdotal observations showed that most birds seemed to visit the dispensers. However, we also noticed that one pullet consistently showed dominant behavior towards pen mates, hindering others to access the larvae. In general, visits were most frequent when fresh dispensers were placed each morning, and declined over time during the day. Often, not all larvae were consumed. The larvae may have become too difficult to reach because they tended to cluster and form a ball, a typical behavior they express in crowded circumstances (Wu et al., 2023). Competition, frustration, or lack of interest in the enrichment provided are risks associated with providing enrichment on a group level. In a future experiment, it would be interesting to link individual interaction with enrichment objects to outcome parameters in individual tests. Another important reason why we did not find convincing evidence might be related to the enriched environment in which all pullets in the present study were reared in terms of high surface area per bird and exposure to daylight. In addition to enriched housing, the pullets were being handled frequently from hatching onwards due to the large number of measurements and behavioral tests performed. Previous studies have shown that handling decreases fear of humans in ISA Brown chicks (Jones, 1994, 1995) Furthermore, the classical music that was played 24/7 to avoid startle responses by sudden noises in the facility could have reduced stress (Davila et al., 2011). All these factors can be considered enrichment, and may have reduced stress responses and fearfulness up to a level that potential effects of larvae enrichment were no longer measurable in the tests performed. In fact, this might also explain the little contrast in fearfulness found between the light- and dark-incubated pullets. Overall, the plasma CORT values measured in the current study were low compared with that reported in the literature, after hatching (Hedlund et al., 2019) as well as at adult age (De Haas et al., 2012; van der Eijk et al., 2019). In addition, the latency to rise in the TIT in our study was also lower than in the literature (Hedlund et al., 2019), as was the latency to struggle in the MRT (van der Eijk et al., 2019). These comparisons indicate that the pullets in our study were less fearful or less sensitive to stressors. Lastly, the randomization error that caused unbalanced treatment groups (see Methods section 2.1) caused a slight power reduction, even though the addition of the extra pens in batch 2 ensured sufficient statistical power to investigate the treatment effects in this study.

## CONCLUSIONS

A green light–dark cycle throughout incubation did not affect CORT or brain calbindin1, DCX, or NeuN levels, but did slightly reduce the fear of humans in laying hen pullets in one behavioral test. However, when considering the number of behavioral tests in which no incubation effect was found, this evidence is insufficient. Larvae provisioning as enrichment did not affect fear behavior or CORT levels in plasma and feathers. Despite the small effects in the current study and given the body of literature that suggests a positive impact of both lighted incubation and enrichment during rearing on behavioral and physiological traits, additional research is recommended to further elucidate the full potential of these strategies to improve laying hen welfare.

## CRediT authorship contribution statement

**Saskia Kliphuis:** Data curation, Formal analysis, Investigation, Methodology, Project administration, Visualization, Writing – original draft. **Maëva W.E. Manet:** Data curation, Formal analysis, Investigation, Methodology, Project administration, Visualization, Writing – original draft. **Vivian C. Goerlich:** Supervision, Writing – review & editing. **Rebecca E. Nordquist:** Supervision, Writing – review & editing. **Hans Vernooij:** Formal analysis, Writing – review & editing. **Frank A.M. Tuyttens:** Conceptualization, Funding acquisition, Writing – review & editing. **T. Bas Rodenburg:** Conceptualization, Funding acquisition, Investigation, Supervision, Writing – review & editing.

## Appendix Supplementary materials

Image, image 1

Image, image 2

Image, image 3

Image, image 4

Image, application 5

## Footnotes

Supplementary material associated with this article can be found in the online version at doi:10.1016/j.psj.2024.103665.
