# Supplementary figures and images for: Effects of lighted incubation and foraging enrichment during rearing on individual fear behavior, corticosterone, and neuroplasticity in laying hen pullets
Source: Poult Sci. 2024 Mar 15;103(6):103665. doi: 10.1016/j.psj.2024.103665 (PMC10999657; doi:10.1016/j.psj.2024.103665)

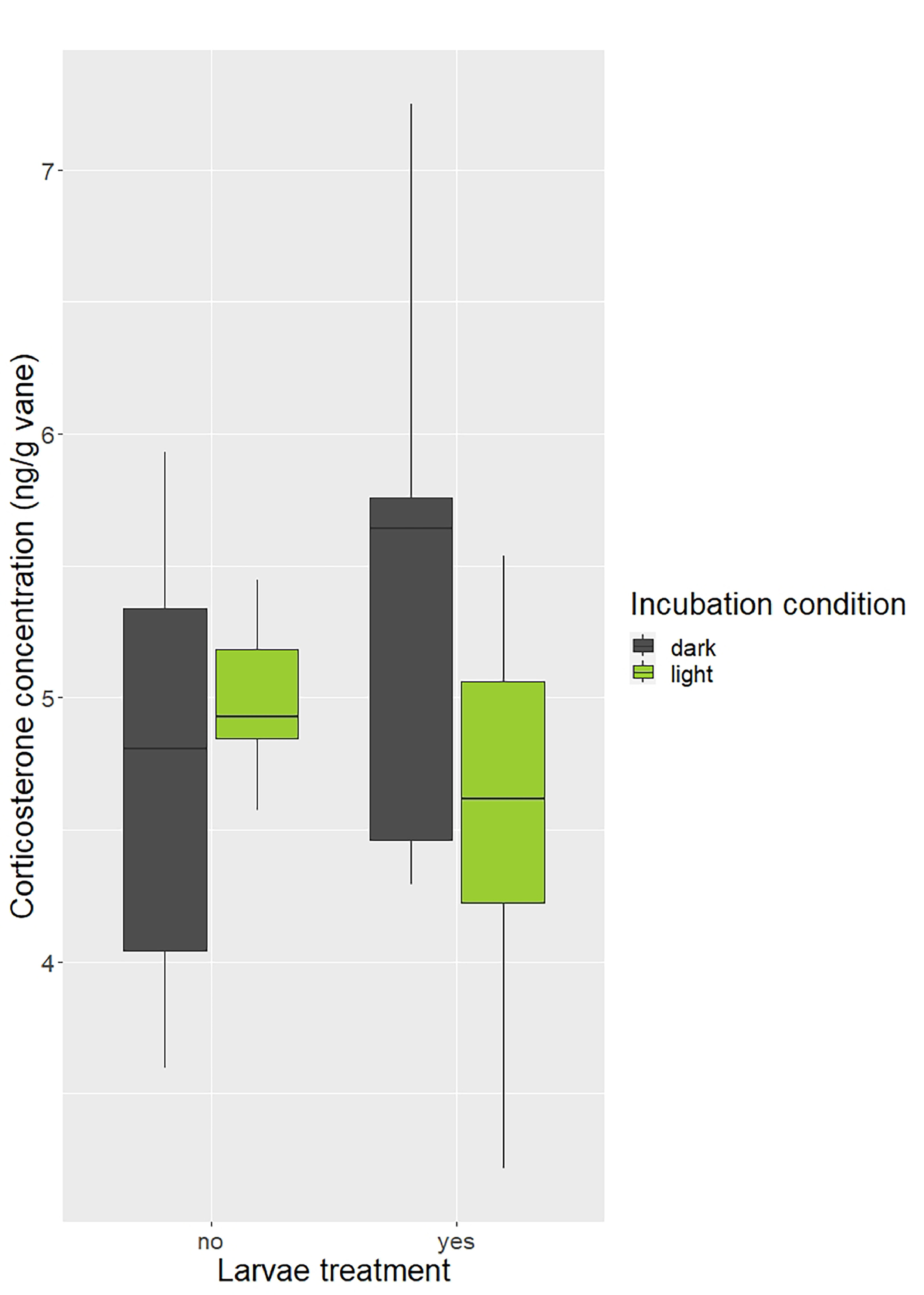

Supplement: Supplementary file 1 [file mmc1.jpg]

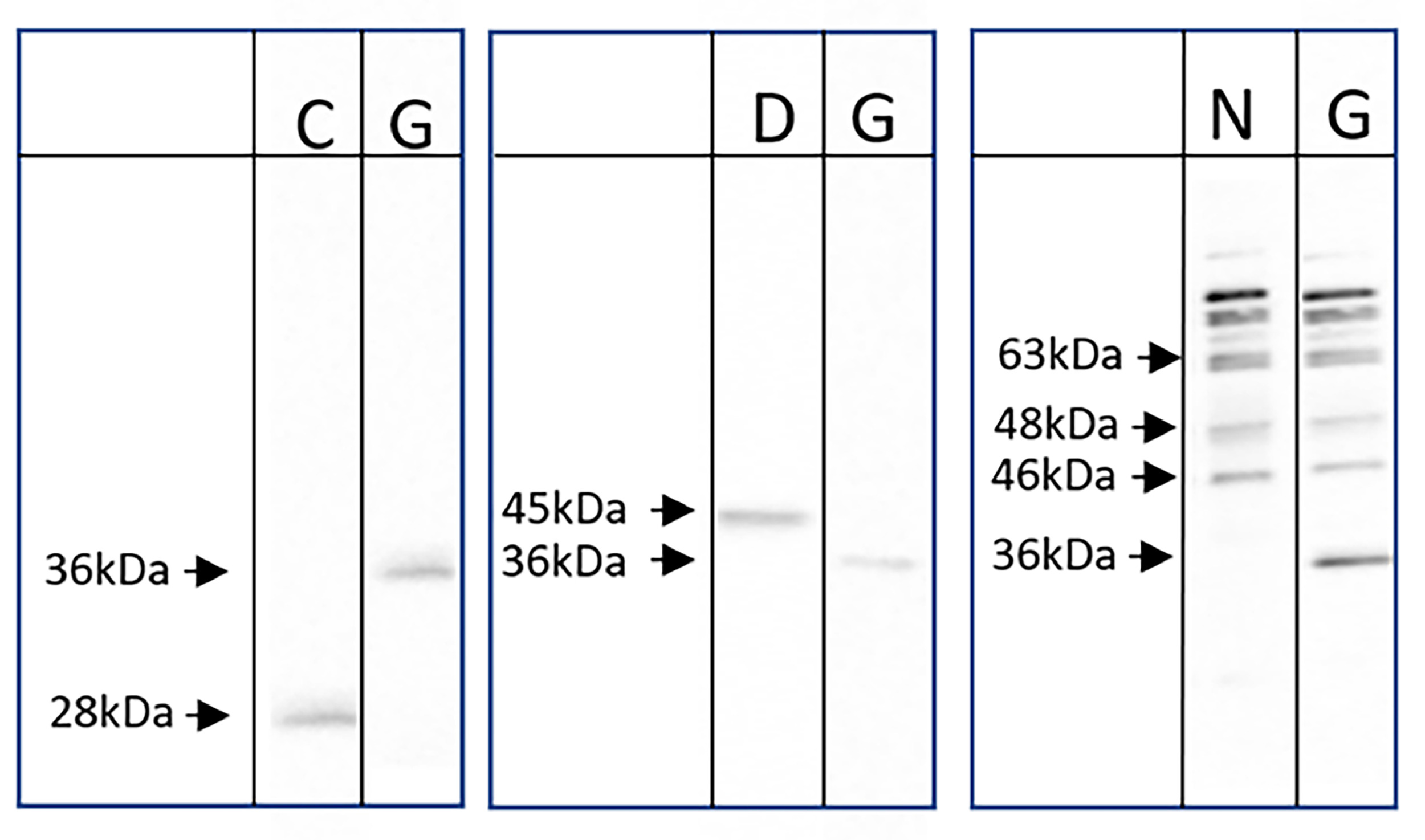

Supplement: Supplementary file 2 [file mmc2.jpg]

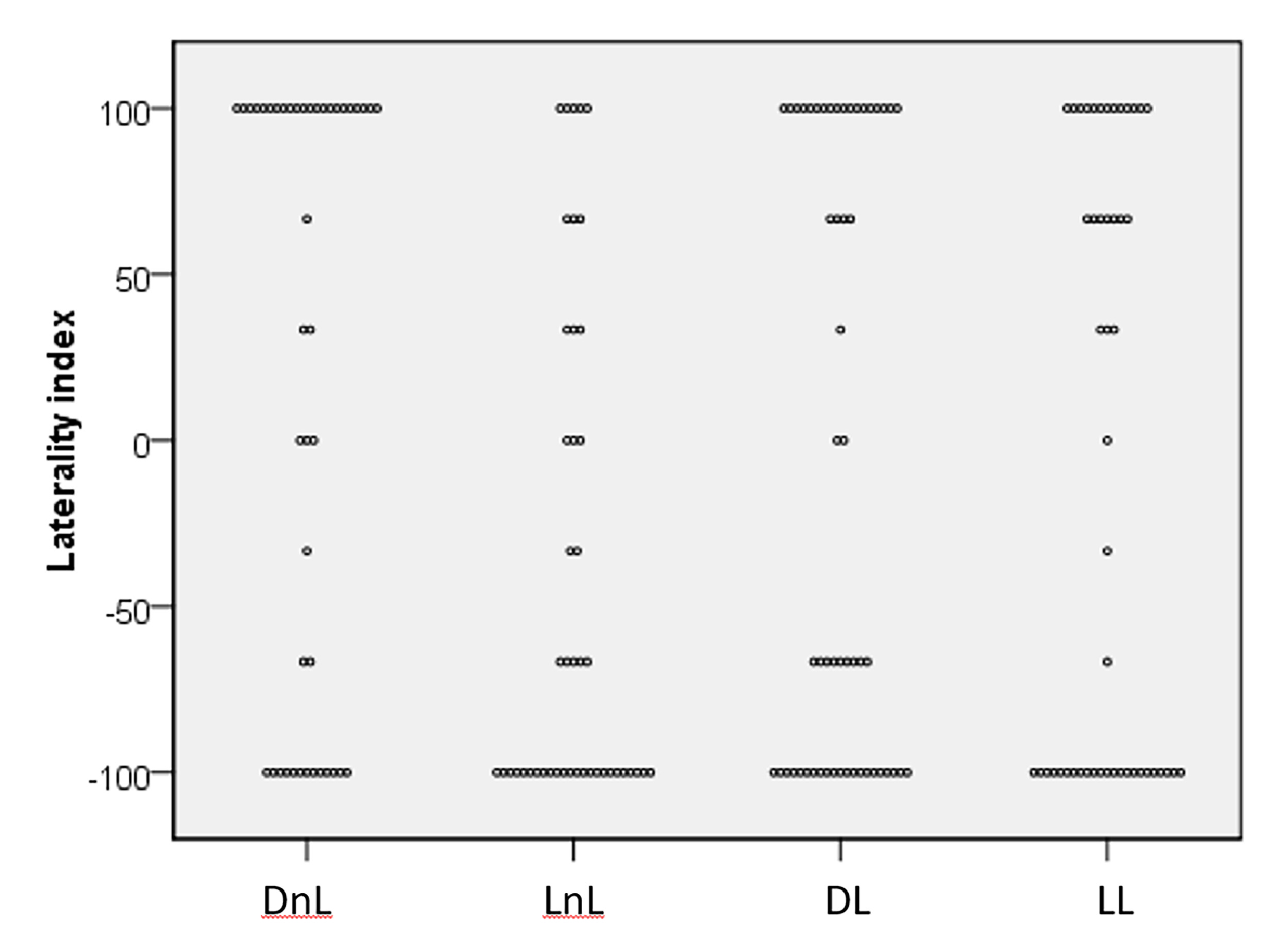

Supplement: Supplementary file 3 [file mmc3.jpg]

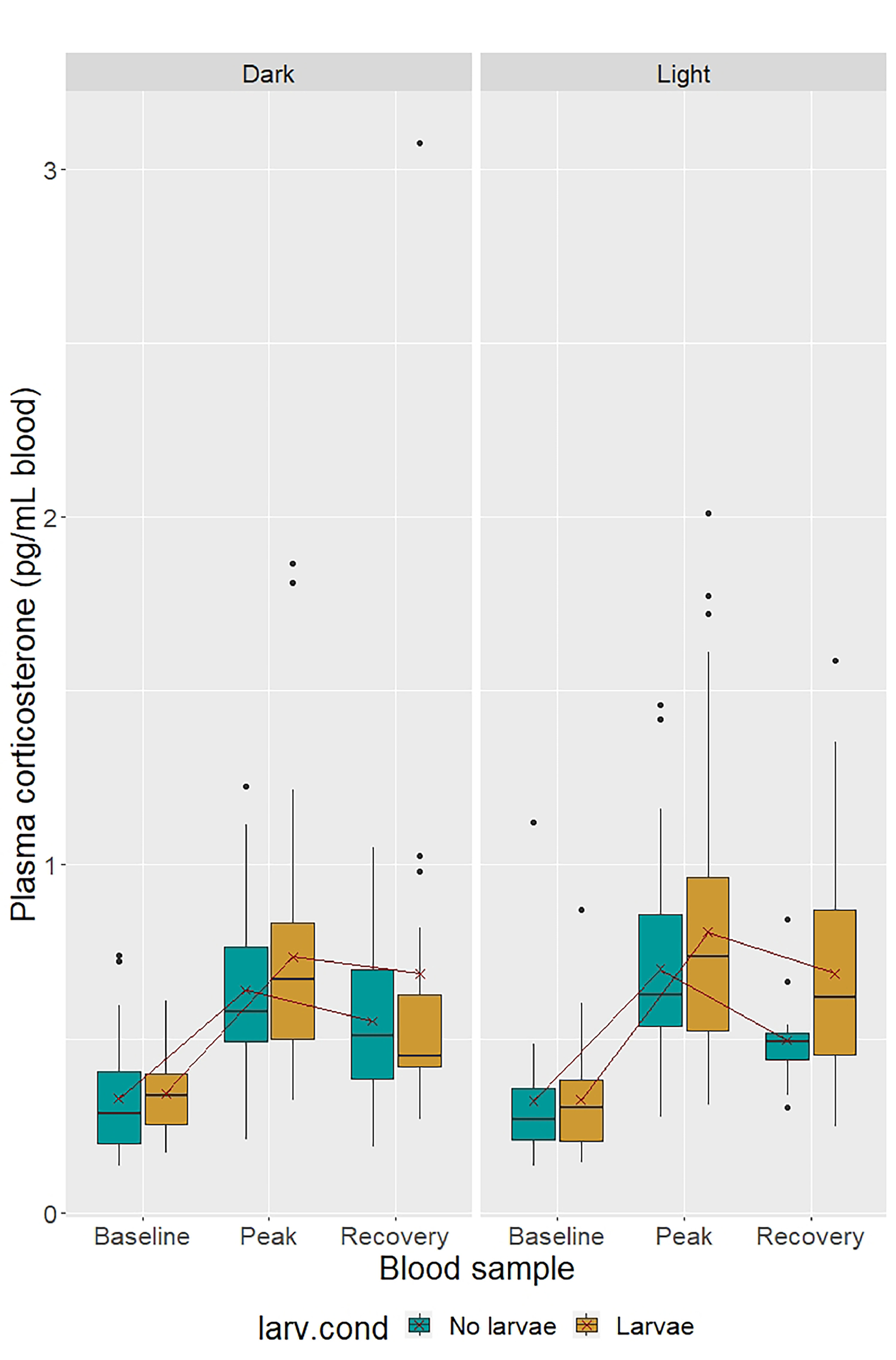

Supplement: Supplementary file 4 [file mmc4.jpg]
