# Supplementary material for: Effects of lighted incubation and foraging enrichment during rearing on individual fear behavior, corticosterone, and neuroplasticity in laying hen pullets
Source: Poult Sci. 2024 Mar 15;103(6):103665. doi: 10.1016/j.psj.2024.103665 (PMC10999657; doi:10.1016/j.psj.2024.103665)

# **SUPPLEMENTARY MATERIALS**

## Supplementary figures

**Figure S1**. Corticosterone concentration in feather 8 of laying hens at 17 weeks of age, for both incubation conditions (dark vs light) and both larvae conditions (yes and no).

**Figure S2.** Typical example of Western blot stainings of laying hen chicks at 1 day of age. Calbindin (C), GAPDH (G), DCX (D) and NeuN (N). The arrows indicate the measured bands. All blots were stripped and reincubated with GAPDH.

**Figure S3**. Laterality index measured during the detour test, performed in laying hen pullets at 3 weeks of age. The laterality index was calculated as ((R-L)/(R+L))*100. Thus, -100 means 100% choice for the left side, 100 means 100% choice for the right side to pass the barrier. Dots represent individual birds. Treatment groups: Dark, no larvae [DnL]; Dark, larvae [DL]; Light, no larvae [LnL]; Light, larvae [LL].

**Figure S4.** Boxplot of plasma corticosterone concentrations during the manual restraint test (MRT) at 15 weeks of age, for laying hen pullets in both incubation conditions (Dark, Light) and both larvae conditions (No larvae, larvae). Samples were taken before (baseline), 15 (peak) and 30 (recovery) minutes after restraint. The X in the box represents the mean.

**Standard Operating Procedures**

### **I. Corticosterone extraction from down feathers from day-old chicks (*Gallus gallus domesticus*) – Utrecht University, Animals in Science & Society, standard operating procedure**

**Chemicals**

| **Chemicals** | **Supplier** | **Product Nr.** |
| --- | --- | --- |
| Methanol | Merck | 1.06009 |
| 1,5 mL tubes | Brand | 780500 |
| 2,0 mL round-bottom tubes | Eppendorf | 0030 120.094 |
| 2mL Micro tube (screw) | Sarstedt | 72.694.106 |

**Materials**

- Beads (Lab Services BV Biospec Products, 3.2 mm no 11079132)
- Tissue Lyser II (Cat. No. 85300, Qiagen)
- Speed Vac Concentrator, Labconco
- End-over-end roller (home-made)

**Procedure**

1. Wear gloves in the whole procedure.
2. Collect and store (-20°C) from down feathers from chicken as described in the relevant protocol.
3. Washing procedure:

Spray the wings with 80% methanol and incubate in a stove at 37°C for 3 days without the use of fan.

1. (Optional: after drying wings can be refrozen at -20°C if necessary.)
2. Pluck the down-feathers from 1 wing.
3. Put the down-feathers (~30-60 mg) in a 2 mL round-bottom tube. Add 3 beads.
4. Use the tissue-lyser 3 x 15 minutes to grind the down-feathers at 30 Hz.
   Centrifuge 1 min. max (~17000 g) between each grind.
5. Weigh exactly the feathers in a 2 mL tube with screw.
6. Add 1000 µL 80% Methanol.
7. Incubate overnight on end-over-end roller.

*(Put 5 tubes in a 50mL Falcon-tube)*

1. Centrifuge max. 5 minutes (~17000g).

Take 600µL and transfer to 1,5 mL tube.

1. Centrifuge max. 5 minutes (~17000g).

Take 2x 200µL and transfer to 2 new 1,5 mL tubes.

1. Dry the methanol from the tubes in a Speed Vac Concentrator (CentriVap Concentrator Labconco) at 42ºC for 1 hour. The lids of the tubes have to be open.
2. Close the lids and store at 4°C.
3. Use the corticosterone Cayman ELISA kit #501320-480 and follow its protocol.
4. Remove the beads from the tubes and wash with the following steps:
   1. Wash the dirty beads in a petri dish with tap water until they are visibly clean
   2. Put the beads in a 50 mL tube and add methanol
   3. Incubate this tube on a gentle mixer overnight
   4. Decant methanol and add pure ethanol
   5. Dry the beads and put them again in a clean petri dish

**Reference**

Davenport, M. D., Tiefenbacher, S., Lutz, C. K., Novak, M. A., & Meyer, J. S. (2006). Analysis of endogenous cortisol concentrations in the hair of rhesus macaques. *General and Comparative Endocrinology*. Volume 147, Issue 3, July 2006, Pages 255-261, ISSN 0016-6480

### **II. Corticosterone extraction from adult flight feathers from laying hens (*Gallus gallus domesticus*) – Utrecht University, Animals in Science & Society, standard operating procedure**

**Procedure**

1. Remove dirt from the feather by gently rubbing with a tweezer.
   In case of bloody feathers, follow the next in-between steps:
   1. Put the feather in an empty 15 mL tube and add 13 mL PBS solution.
   2. Shake the tube for 10 minutes.
   3. Rinse the feathers once more in the PBS solution.
   4. Wash the feathers with milliQ to get rid of the PBS solution.
   5. Dry the feathers in the stove at 37 °C for 1 hour.
2. Put left and right feathers together on a paper/foil.
   Indicate which is left and which is right.
3. Note the irregularities of the feathers (*e.g.* fault bars, loss of barb/barbule).
4. Take a photograph of each feather including a scale-bar and the chicken ID on a contrast (one color) background.
5. Wash the feathers with 100% methanol in the fume hood: quickly spray both sides of the feather (stick with tape on the calamus ) and dry the feather in the fume hood on a piece of paper.
6. Measure the length of the feather without calamus (now or at point 8).
7. Remove the calamus (including downy barbs).
8. Measure the length of the feather without calamus (now or at point 6).
9. Weigh the feather, rounded to mg (scale: 0,0001 g exactly).
10. Collect the vanes by cutting next to the rachis from the tip to the calamus.

(Optional: Cut the vanes on colored paper into +/-7 mm pieces in order to get them in the tube).

1. Weigh the rachis, rounded to mg (scale: 0,0001 g exactly) and calculate the weight of the vanes (feather weight – rachis weight).
2. Transfer the vanes into 2,0 mL round bottom-tubes.
   Weight in 0,02 g +/- 0,002 g vanes per tube.
   Note the exact weight.
   Add 3 beads per tube.
   Repeat this until all the vanes are in tubes. The final tube will probably differ in weight. Number the tubes consecutively. (Consider pooling left and right with the same feather numbers if your research question allows it)

*(Take as many tubes as necessary to insert the feathers and to grind well).*

1. Use the tissue-lyser 3 x 15 minutes to grind the feathers, 30 Hz.
   Centrifuge 1 minute max (~17000 g) between each grind.
   Store all your tubes at this point until you’re ready for extraction.
2. Centrifuge the tubes for 1 minute 14000 rpm (1500 g).
3. Pipet 1,0 mL methanol 100% per tube.
4. Wrap up the tubes lightproof (to avoid corticosterone degradation) and place the tubes overnight on an end-over-end roller (speed 30).
5. Centrifuge the tubes for 1 minute 14000 rpm (1500 g).
6. Transfer 700 µL of each of the Eppendorf tubes into one 15 mL tube (Heraeus Multifuge X3R, Thermo Fisher Scientific, Langenselbold, Germany) so you collect the supernatants of the probably 5 or 6 different tubes containing the vanes of the same feather.
7. Take 2x 1200 µL and transfer to 2x 1,5 mL tube. Keep the left-overs for step 21.
8. Centrifuge max. 5 minutes (~17000 g).

Take 2x 800 µL and transfer to 2 new 1,5 mL tubes.

Dissolve in 160 µL ELISA buffer.

1. Pool an equal amount of the left-overs from step 19. Centrifuge as in step 18 and make aliquots of exactly 1000 µL as controls for try outs and plate controls.
2. Dry the methanol from the tubes in a Speed Vac Concentrator (CentriVap Concentrator Labconco) at 42ºC for 1 hour. The lids of the tubes have to be open.
3. Store at 4°C until use.
4. Use the corticosterone Cayman ELISA kit #501320-480 and follow its protocol.

Extra tips:

- Wash your scissors with 70% ethanol and change gloves after cutting a feather (or more, if you put more feathers into one tube).
- Use colored paper to cut on, this helps you see all the small feather parts
- Cut the feathers in the spring or summer time, when you need to cut in the autumn of winter: place a water bath next to you that will make the air in the lab more humid. By doing this, the feathers will be less static and it will help you with cutting them

<https://biospec.com/beads-guide-lines/cleaning-your-beads>

**References**

Nikole E. Freeman and Amy E. M. Newman (2018): Quantifying corticosterone in feathers: validations for an emerging technique

Bortolotti et al (2009): Tracking stress: localisation, deposition and stability of corticosterone in feathers

K. E. Häffelin et al. (2020): Corticosterone in feathers of laying hens: an assay validation for evidence-based assessment of animal welfare

P. Møller et al. (2009): Frequency of fault bars in feathers of birds and susceptibility to predation

### **III. Corticosterone extraction from (chicken) plasma – Utrecht University, Animals in Science & Society, standard operating procedure**

**Chemicals**

| **Chemicals** | **Supplier** | **Product Nr.** |
| --- | --- | --- |
| Dichloromethane | Lab scn analytical sciences |  |
| Optional: ELISA buffer from ELISA anti corticosterone | Cayman | #501320-480 |

**Materials**

Brand 1,5 ml vials

12,5 ml pipet tip (repeater pipet)

Multivortex

Eppendorff tabletop centrifuge

Certrivab conentrator (labonco)

**4. Solutions**

**-**

**5. Procedures**

1. Thaw plasma sample
2. Pipet 100µl plasma into clean vial
3. Add 400µl Dichloromethane using the 12,5 ml repeater pipet
4. Vortex 15 min on 6
5. Centrifuge 3 min at 13000rpm
6. Collect organic extract (2x200µl) in clean vial; evaporate dissolvent in centrivap at 42 degrees C; repeat steps 3-5 another 2 times and collect the extract in the same tube.
7. Evaporate completely and store dry at -20degrees C

Chicken plasma samples of ISA Brown and White Leghorn chicken (PPILOW and ChickenStress projects ) were dissolved in ELISA buffer provided with the Cayman kit (#501320; 400µl) by shaking at 50 degrees C for 30 min.

**References:**

**Cayman kit booklet (**(#501320) <https://cdn.caymanchem.com/cdn/insert/501320.pdf>

### **IV. Processing samples and Quantative SDS-PAGE/Westernblot – Utrecht University, Animals in Science & Society, standard operating procedure**

**1. Introduction:**

Polyacrylamide gels are formed by the reaction of acrylamide and bis-acrylamide (*N,N*’-methylenebisacrylamide) that results in highly cross-linked gel matrix. The gel acts as a sieve through which the proteins move in response to the electric field. Proteins contain an overall positive or negative charge; this enables the movement of a protein molecule towards the isoelectric point at which the molecule has no net charge. The ionic detergent SDS denatures and binds to proteins and giving them a uniform negative charge, it is possible to separate them based on the size as they migrate towards the positive electrode.

**2. Chemicals**

| **Chemicals** | **Supplier** | **Product Nr.** |
| --- | --- | --- |
| Stainless Steel beads (3,2mm) | Biospec | 11079132ss |
| DC protein Assay Kit II (inclusive standard) | Bio-rad | #500-0112 |
| RIPA buffer | Thermo Fisher | #89900(100ml) |
| Protease and phosphatase inhibitor (PP) | Thermo Fisher | 78440 |
| 4x Laemmli sample buffer | Self-made |  |
| β-mercaptoethanol | Merck | #01496DG |
| Tris/Glycine/SDS Electrophoresis buffer | Bio-rad | #161-0732 |
| Blot buffer | Bio-rad | #161-0734 |
| Methanol | Merck | #106009 |
| Washing buffer, TBST | Bio-rad | #170-6435 |
| Tween | Biorad | #170-6531 |
| Thick Blot Filter Paper | Biorad | #1703932 |
| Nitrocellulose membrane | Biorad | #162-0115 |
| Milchpulver Blotting grade fet-arm | Roth | T145.2 |
| Anti- NeuN | Merckmillipore | MAB377 |
| Anti-Double cortin | Abcam | ab18723 |
| Anti-Calbindin D28k | Swant | CB28 |
| Anti GAPDH 1:60000 | Proteintech | 60004_1-1g |
| GARPO (Goat-anti-rabbit) Pierce biotechnology 1:10000 | Thermo | #31460 |
| GAMPO (Goat-anti-mouse) Santa Cruz 1:10000 | Abcam | #ab6789 |
| Supersignal West Dura Extended duration Substrate | Thermo | #34075 |
| Criterion TGX precast gels 8-16% (bio-rad) 15µl | Bio-rad | #5671105 |
| Abcam prism ultra protein Ladder | Abcam | #AB116028 |

**3. Materials**

Analytical balance AC210P (Sartorius)

Tissuelyser II (Qiagen)

Centrifuge(

Vortex(Scientific industries)

Thermoblock(Eppendorf)

SDS-PAGE and Blot system(Biorad)

Chemidoc (Biorad)

**3. Procedures**

**Preparation of frozen (brain) tissue**

Use 2 nights to get to the desired temperature of the tissue, from -80 to -20 and

-20 to -4°C.

- Cold blocks (out of the -20°C)
- Aluminium plates
- (Dry) ice
- Weigh (Ohaus) and mark (cryo marker) the vials (2 ml)
- Pincers
- Spatulas small
- Gloves

Get the desired part out of the brain, and put in the cooled vial, weigh and put on ice. If not used the same day, store in the -80 ̊C.

**Homogenizing protocol**

- Centrifuge
- Tissuelyser
- Beads
- RIPA
- Protease and Phosphatase inhibitor P (10µl/ml RIPA)
- Gloves

1. (Write vials (5x’ 2ml-eppendorf round bottom tubes (per sample))
2. Add beads to the tissue (3/ep, RIPA rinsed)
3. Add RIPA-buffer +PP (10 µl/1mg tissue) (or added ¼ RIPA when amount too large)
4. Put 8x20seconds, 30Hz in the Tissuelyser (Qiagen) (2 minutes on ice in between each cycle)
5. Spin 10 sec 2000rpm 4°C
6. Make aliquots of 200µl in 2,0ml tubes.
7. Store at -80°C

To continue

1. Continue with 1 aliquot.
2. Add 600µl RIPA +PP +1/4 PP (optional: if at point c is added ¼ RIPA )
3. Add 3 beads to the sample and tissuelyse 2 x 20 seconds, 30Hz in the tissuelyser.
4. Centrifuge 2min. 2000rpm and transfer supernatant to 1,5 ml tube. (keep on ice)
5. Measure the protein concentration.
6. Bring the concentration to 200µg/30 µl with RIPA+ PP and transfer 30µl to a 1,5 ml tube.
7. Store aliquots at -20°C.

**Protein determination according to Bio-Rad:**

- Reagent A #500-0113
- Reagent B #500-0114
- Reagent S #500-0115
- Ampule Pierce BSA 2 mg/ml NaCl
- 96-wells plate
- Plate reader
- Atomizer with ethanol

1. Make a work reagent; add 20µl S to 1 ml A => A* (for total plate 2.2 ml A* required)
2. Make a dilution series of BSA (0.2 -2.0 mg/ml), dilute with the same solution as you diluted the samples = standard.
3. 100µl **Ampule Pierce** + 0µl **RIPA** 2 mg
4. 75µl ,, + 25µl ,, 1.5 mg
5. 50µl ,, + 50µl ,, 1.0 mg
6. 37.5µl ,, + 62.5µl ,, 0.75 mg
7. 25µl ,, + 75µl ,, 0.5 mg
8. 10µl ,, + 90µl ,, 0.2 mg
9. 0µl ,, + 100µl ,, 0
10. Extra included 100µl NaCl
11. 5µl standard (duple) or sample (triple) in 96-wells plate, flat bottom.
12. Add 25µl A* to each well.
13. Add 200µl B to each well, to remove the bubbles use the atomizer (spray with 70%EtOH).
14. Plate reader, shake 5 seconds, after 5-15 minutes read at 650nm and 405nm, shake in between, absorbance is stable for 1 hr. *we did 2 runs (5’, 30’) and choose the 2^nd^*

If unknown, dilute a few samples 5x, 10x, 25x, 50x and 100x, and determine the correct dilution for all the samples.

For the 1-day old chicks, left hemisphere we use 5x and 10x.

**4. Solutions SDS-PAGE/ Westernblot**

**Stocks:**

**Electrophoresis buffer  (10xTris/Glycine/SDS buffer**

**#1610732**

To make 1 L of working buffer add 100 ml buffer concentrate to 900 ml MilliQ. Mix thoroughly. Final concentration of a 1x solution is 25mM Tris. 192 mM Glycine and 0,1%(w/v) SDS. pH 8.3. Do not adjust pH.

**Transferbuffer (10xTris/Glycine buffer=>Blot buffer)**

**#1610734**

To make 1 L of working buffer add 100 ml buffer concentrate to 200ml methanol and 700ml milliQ. Mix thoroughly. Final concentration of a 1x solution is 25mM Tris. 192 mM Glycine, and 20% (w/v) methanol, pH8.3.

Do not adjust pH. (Makes 10 lL of working solution. For running native gels omit methanol from above recipe.)

**10xTBS buffer=> 1x TBS buffer**

**#170-6435**

Dilute 100ml of 10X TBS with milliQ to make a solution of 20mM Tris, 500mM sodiumchloride, pH 7.5

**Stripping reagent TBST+ 0,02% NaAz**

0,02g per 100ml TBST

**-Wash buffer (TBST) #170-6435,**

100 ml (10X) + 900 ml MilliQ + 0.1% Tween-20 (1ml Tween20/1000ml TBST)

**-Block Buffer with 5% non-fat dry Milk (use within 1 wk)**

5,0 g non-fat dry milk in 100 ml TBST

**-Block Buffer +0,02% NaAz with 5% non-fat dry Milk (use within 1 wk)**

5,0 g non-fat dry milk in 100 ml TBST + 0,02% NaAz, *rollerbank*

Storage (except for non-diluted electrophore and blot buffer) at 4ºC.

**5. SDS-PAGE/ Westernblot**

Protocols for buffers and sample dilution can be found at **point 4** of this protocol.

*Sample preparation*: (Keep samples on ice!!!!!)

defreeze samples on ice (200µg/30µl)

1. Heat WB Heating block to 100°C
2. Leave samples on ice as long as possible
3. Make Sample Buffer (900µl 4x Laemmli + 100µl βME) in hood, vortex => SB_βME_
4. Prepare samples, calculate to volume of 10µl using pre-made calculations.
5. Add per sample 170µl (50µl SB_βME_ + 120µl RIPA), Final concentration is 10µg/10µl
6. Place STOCK samples in Heating Block (100°C) for 7 min. (Normally 5 min. ~50 µl)
7. Place directly on ice

*Electrophorese:*

1. Open ready gel according to the leaflet and remove comb (top)
2. Place gel in gel-container (large side in front, red left), match ridge on glass with rubber ridge on holder.
3. Add running buffer to middle compartment (above wells),be sure there is no leakage.
4. Fill rest of container with running buffer (at least about ¼ of the tank).
5. Rinse the wells with running buffer.
6. Load ladder: Abcam prism ultra-protein Ladder **7.*5µl will be enough?!***
7. Load samples: 10μl per sample.
8. Place lid on container: match red to red; black to black.
9. Place container on ice to prevent uneven pulling of the samples (resulting in smileys).
10. Start gel electrophoreses: Bio-Rad Power Pac.

**Use constant Voltage 200V, guideline 60-100min.**

*Blotting:*

**Take care!!!! The Criterion gel is very fragile. Remove the wells on the plate.**

1. Prepare blot buffer (place in cold room until right before use).
2. Take a box (preparation of blot), blot holders, filter paper (size of membrane), sponges, membrane (nitrocellulose; do not touch with bare hands and hold by edges, preferably with forceps.
3. Make your transfer package, ensure there are no bubbles between the layers!: 10 min prior to use.

Soak in blot buffer.

- - - Take blot holder and place it in the box filled with blot buffer (black side down).
    - Soak sponge, place it on the blot holder.
    - Soak filter paper, place it on the sponge.
    - Take gel out of glass plates, and remove a small corner where the ladder is.
    - Place gel on filter paper (upside down).
    - Soak membrane, cut of top right corner, and place on top of gel (upside down).
    - Soak filter paper, place on membrane.
    - Soak sponge, place on top of filter paper.
    - Close your blot holder and place it in the tank (black side of holder facing the black side of the container).
    - Put ice container and stirrer in.
    - Fill the tank with blot buffer and place on ice on a magnetic plate.
    - Place lid on container: match red to red; black to black.
    - Run 100 V 1 hour ± 190 mA.

**Use the following schematic for building the blot: sandwich**

- - White = anode

**Negative**

Positive

Gel

Blot

- - Sponge
  - 1x filter paper
  - Membrane(blot)
  - Gel
  - 1x filter paper
  - sponge
  - Black = Cathode

Remark: after blotting is top right top left

Blotting system

**
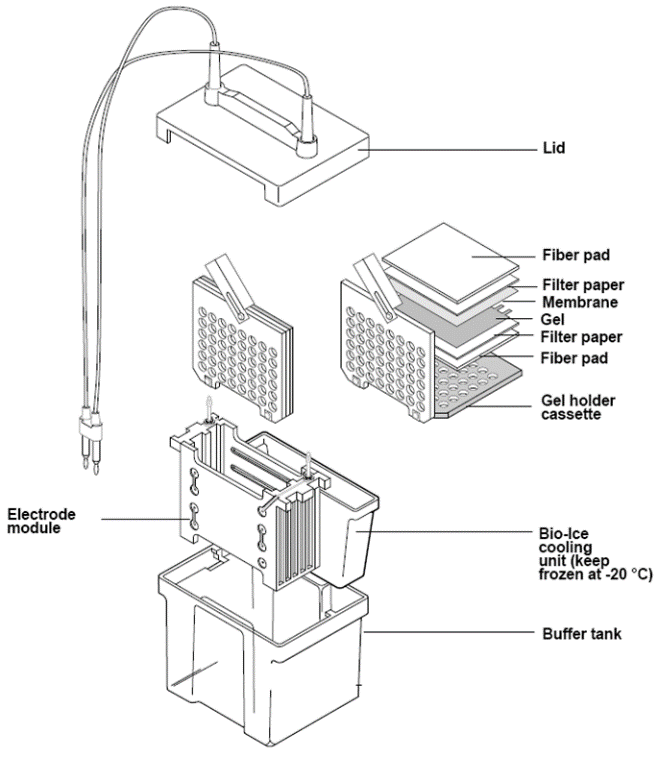
**


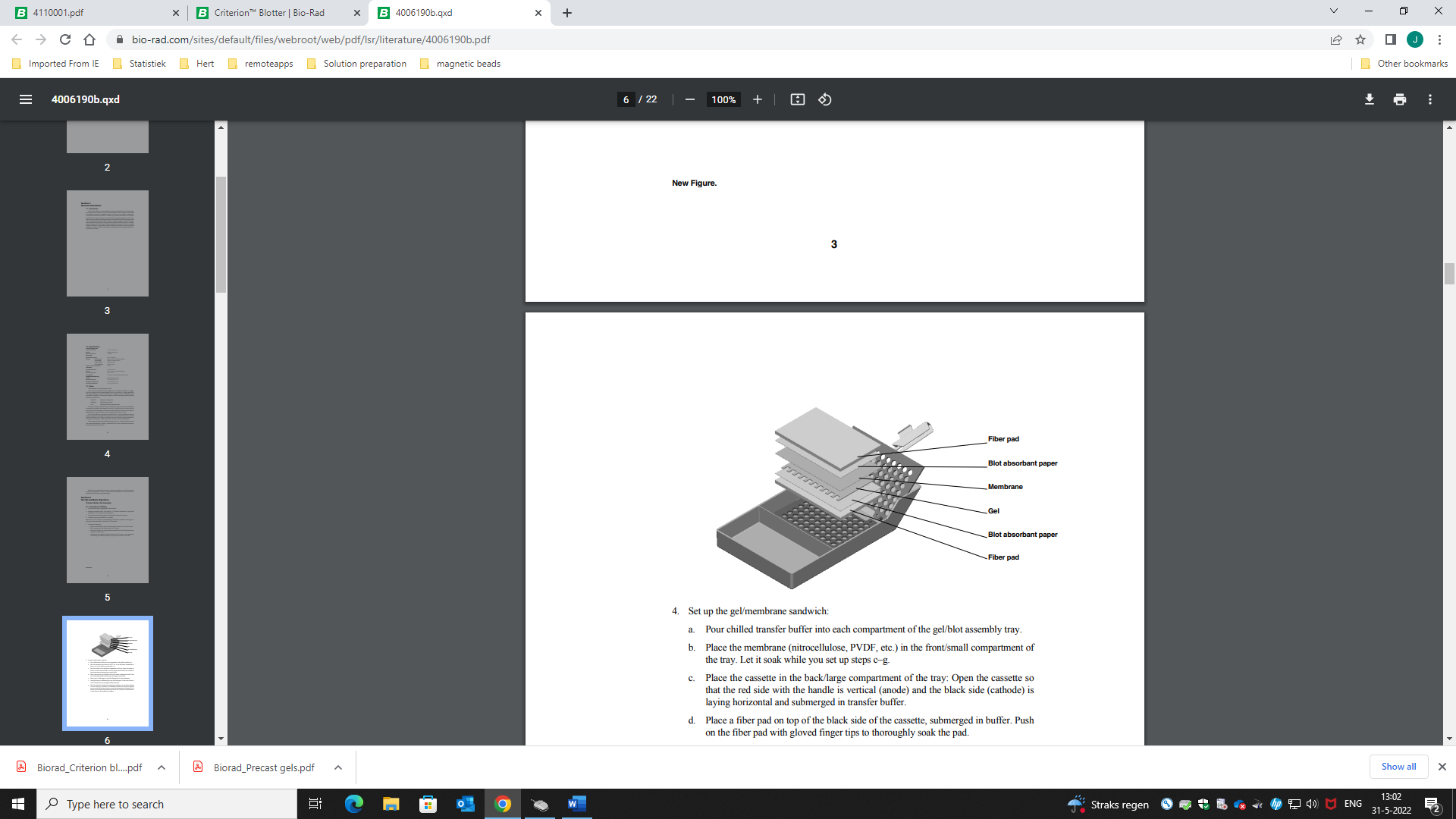


**(Immuno)staining:**

Midi blot 13.3 × 8.7 cm_use 15ml (in box of 14x18cm)

*Day 1*

1. Prepare blocking buffer: TBST (*wash buffer)* + 5% non-fat dry milk. *Block buffer on stirrer/rollerbank for at least 1 hr*
2. Take membrane out of package and place in a box, with the ladder/samples facing upwards.
3. Wash 1x5 min with wash buffer at roomtemperature (T_R)_, shaker speed 30 (20ml)

Wash with TBS and TBST

1. Block the membrane in block buffer 1h at T_R_, shaker speed 30 (20ml)
2. Prepare primary antibodies in block buffer: 15’ in advance
   1. Mouse anti NeuN 1:1000 (46-48 kDa)

(Don’t use 5% milk in the blockbuffer)

- 1. Rabbit anti DCX 1: 1500 (45 kDa)
  2. Rabbit anti Calbindin 1:1000 (28kD)
  3. Mouse anti GAPDH 1:60000 (36kD)

1. Incubate with 15 ml primary antibodies, overnight (ON) at 4°C speed 30

*Day 2*

1. Wash 3x5 min with wash buffer on shaker speed 30
2. Prepare secondary AB in block buffer 15’ in advance 1:10.000
   1. Goat anti-rabbit peroxidase GARPO
   2. Goat anti mouse peroxidase GAMPO
3. Incubate secondary AB for 1h at RT
4. Wash 4x10 min with TBST and 1x10 min TBS on shaker
5. transfer in TBS to biochemistry - ChemiDoc, take with you, Supersignal West Dura Extended duration Substrate, pincers, sheets, stopwatch, gloves.
6. E.g. blot in 14x18cm box- use 3 ml white bottle+ 3ml brown bottle. (can be re-used for a second blot within 1 hr).
7. Incubate 5min. in dark, and swerve regularly
8. Put the blot on 1 sheet, to prevent contamination of the chemidoc.
9. scan blots

**For soft stripping:**

Wash with TBS, to remove the ECL.

Wash with TBST

Incubate 1 hr with TBST +0,02% NaAz.

Incubate 1 hr with TBST +0,02% NaAz.+5% milk

(NaAz can be used as a soft stripping method, It destroys the second antibody with horse radish peroxidase).

1. Prepare primary antibodies in block buffer +0,02% NaAz: 15’ in advance
   1. Mouse anti GAPDH 1:60000 (36kD)
2. Incubate with 15 ml primary antibodies, ON at 4°C speed 30

*Day 3*

1. Wash 3x5 min with wash buffer on shaker speed 30
2. Prepare secondary AB in block buffer 15’ in advance 1:10.000
   1. Goat anti mouse peroxidase GAMPO
3. Incubate secondary AB for 1h at RT
4. Wash 4x10 min with TBST and 1x10 min TBS on shaker
5. transfer in TBS to biochemistry - ChemiDoc, take with you, ECL components, pincers, sheets, stopwatch, gloves.
6. E.g. large blot in 14x18cm box- use 3 ml white bottle+ 3ml brown bottle.
7. Incubate 5min. in dark, and swerve regularly
8. Put the blot on 1 sheet, to prevent contamination of the chemidoc.
9. Scan blots

**Protocol how to make pictures with ChemiDoc (Image Lab)**

**Take a picture of the marker**

- Open the drawer of the ChemiDoc and place the blot in the middle of the glass plate

- Open ImageLab

- 'new protocol' is automatically opened**.**

If not, click on 'New Protocol' at the top left and choose 'Single Channel'.

- Click on 'select'

- Choose 'blots'

- Choose 'colormetics'

- Choose 'biorad ready gel'

- Choose 'The software will optimize the exposure time for' and choose 'faint bands'

- Set highlights by ticking 'highlight saturated pixels'. If there is a red highlight on the strap, the strap is too bright for quantification, and there should be a shorter exposure.

- Click on 'position gel'

- Open the door and place the blot in the middle of the image

- Click on 'run protocol'

**Take a picture of the protein**

*High resolution*

A high resolution photo is used to quantify, because the correlation is sharper - which gives sharper straps.

- Go back to the protocol

- Click on 'select'

- Choose 'Blots'

- Select 'High Resolution'

- Select 'Manually set exposure time' and enter how long the photo needs to be lit up. This differs per protein and blot

- Click on 'Run Protocol'

Pay attention! Do not move the blot! Then you can’t put the photos over each other later.

**Merge photos**

- Open the two photos to be merged (the marker photo and the best protein photo)

- Click on Image tools (Link in the analysis tool box)

- Click on 'Merge'

- Select the image to be merged.

- Click 'ok'

- Save the merged photo.

**Quantifying:**

Use ImageJ to quantify.

According protocol:

http://rsb.info.nih.gov/ij/docs/menus/analyze.html#gels

**!!!For quantification use the picture without merging with the picture of the ladder.**

**Otherwise influence of the lamp from the picture with ladder (colormetric measurement).**

**To Optimize the procedure:**

Check per antibody concentration if the amount of protein is measured in the linear part.

5µg protein/10µl sample, 10µg/10µl, 15µg/10µl, 20µg/10µl, 25µg/10µl.

**Ladder information:**

Use the first marker based on Tris-Glycerin.


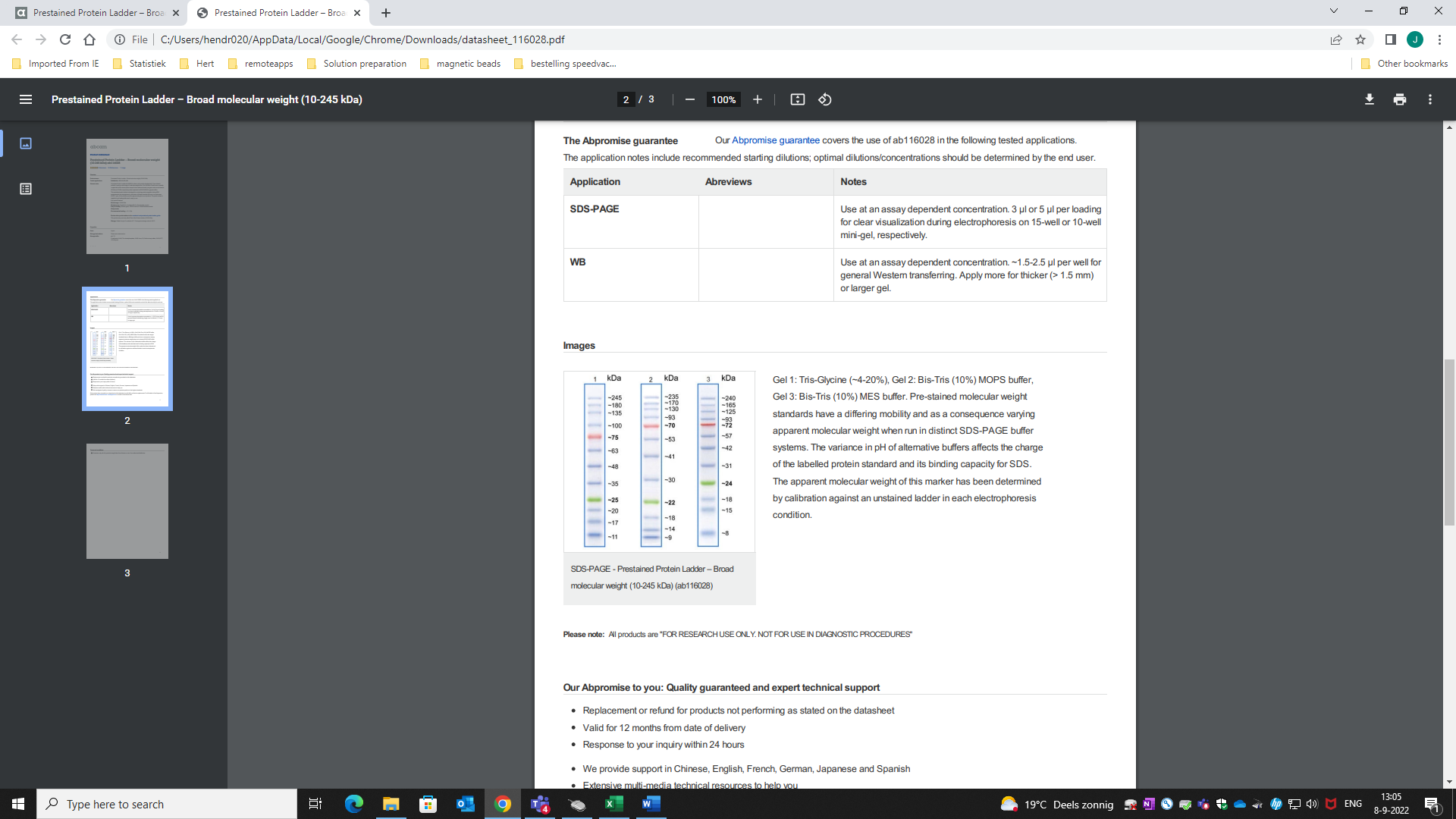

Supplement: Supplementary file 5 [file mmc5.docx]
